# Childhood adversities and prior involvement with child protective services

**DOI:** 10.1186/s40621-019-0224-9

**Published:** 2019-12-09

**Authors:** Shakira F. Suglia, Erin R. Kulick, Jocelyn Brown

**Affiliations:** 1 Department of Epidemiology, Rollins School of Public Health, Emory University, 1518 Clifton Rd, Atlanta, GA 30322 USA; 2 Department of Epidemiology, Mailman School of Public Health, Columbia University, New York, NY USA; 3 Department of Epidemiology, Brown University, Providence, RI USA; 4 Department of Pediatrics, Columbia University Medical Center, New York, NY USA

**Keywords:** Child maltreatment, Re-victimization, Child protective services, Adverse child experiences, Social stressors

## Abstract

**Objectives:**

We aimed to determine the relation between childhood adversities and prior involvement with Child Protective Services (CPS) history among children presenting for evaluation at a Child Advocacy Center.

**Study design:**

The study evaluated children presenting to a Child Advocacy Center (CAC) from 2009 to 2014. A five-item child adversity measure, based on mother’s report, was characterized into a scale of none, one, or two or more adversities. Caseworkers at the CAC assessed whether families had a prior history of involvement with CPS.

**Results:**

Among the 727 children included in the analyses, 43% had a prior history of involvement with CPS. Twenty-six percent of the children experienced one childhood adversity while 29% experienced two or more. In regression analyses adjusting for socio-demographics, experiencing one (Prevalence Ratio (PR) 1.25 95%CI 1.0–1.5) or two or more adversities (PR1.67 95%CI 1.4, 2.0) was associated with higher prevalence of CPS history compared to those who reported none.

**Conclusions:**

Childhood adversities are associated with prior contact with CPS, suggesting there are missed opportunities to provide services to high-risk families. CACs may be in a unique position to advocate for families and prevent further victimization of children.

## Introduction

Childhood maltreatment is a toxic stressor prevalent in the United States (US) (Shonkoff and Garner 2012) that often co-occurs with other adversities, including parental substance use, parental psychopathology, divorce, and other forms of violence exposure (Connell et al. 2007). However, these adverse experiences, commonly known as adverse child experiences or ACEs, are not consistently assessed in clinical settings, including emergency room departments or child advocacy centers (CAC) were children who are victims of maltreatment might be presenting (Campbell et al. 2019; Bethell et al. 2016). During child abuse investigations the focus is generally on the evaluation of maltreatment and often other adversities that families may be facing are not addressed (Campbell et al. 2019). Families who are facing multiple adversities are the most vulnerable, often having low economic, social and emotional resources and thus children may be most at risk for repeat victimization (Connell et al. 2007), identifying and addressing these factors when children are first involved with clinical settings, particularly for suspicion of abuse, may prevent repeat victimization and facilitate prompt referrals to trauma-informed mental health care.

We examined the prevalence of adversities among children seen at a CAC for suspicion of child abuse and their relation to prior involvement with Child Protective Services (CPS).

## Methods

We extracted medical record data from children seen at the Manhattan Child Advocacy Center (MCAC) from February 2009 to November 2014 for suspicion of exposure to sexual and/or physical abuse. The MCAC brings together professionals and agencies as a multidisciplinary team to investigate physical and sexual abuse cases and coordinate services to children and their families (Cross et al. 2007). Specifically, representatives from CPS, law enforcement, district attorney’s office, Safe Horizon (a national victim assistance organization) and a medical team are involved. Children and their caretakers are referred for further investigation and medical assessment after a child abuse report is made to law enforcement and/or CPS. Certain criteria for evaluation by the multidisciplinary team at the MCAC must be met: familial or non-familial sexual offenses of children 12 years and under, physical abuse of children 10 years and under, the family resides in New York County and /or the alleged criminal activity occurred in New York County. Children are first interviewed forensically by Safe Horizon trained professionals. The purpose of every forensic interview conducted at the MCAC is to obtain a statement from a child, in a developmentally, age appropriate and culturally sensitive, unbiased and fact-finding manner that will support informed and fair decision making by the Manhattan multidisciplinary team. A medical examination by a board certified child abuse pediatrician is also conducted in about half of the children interviewed. Lastly, the caretaker, usually the non-offending parent is also interviewed by the child abuse pediatrician. Mandated reporting laws suggest that a report is made based on suspicion of abuse, not on definitive proof of abuse. It is then possible that there was no evidence of abuse or neglect after an investigation and therefore that there may not be any abuse.

Of 1069 children who presented at MCAC between 2009 and 2014 and were referred for medical evaluation, 727 were interviewed forensically by Safe Horizon trained professionals and the caretaker, usually the non-offending parent, was also interviewed by the child abuse pediatrician. During the medical assessment, the mother was interviewed alone and asked about past history of mental illness, her own history of incarceration or arrest, drug or alcohol use and history of intimate partner violence (IPV). To assess IPV, mothers were asked whether someone in the past year kicked, hit or punched them and whether in a past relationship they felt afraid or were harmed by their partner (Basile et al. 2007). Presence of an ongoing custody battle was also assessed to define parental discord. Caseworkers obtained access to prior CPS reports. The study was approved by the Institutional Review Board at Columbia University.

### Data analyses

Bivariate analyses were conducted to assess the relationship between child and maternal characteristics and adversities with prior CPS history. Given the high prevalence of CPS involvement, binomial regression analyses were conducted to estimate the association between adversities and CPS involvement. Unadjusted analyses were first conducted followed by a regression model adjusting for child characteristics (race/ethnicity, gender, age, and type of abuse), mother characteristics (age, primary language and education), and receipt of public assistance. All analyses were conducted in SAS 9.3.

## Results

The adversities’ prevalence was high; 32% of mothers reported one adversity and 30% reported two or more. A higher prevalence of adversities was associated with prior CPS report (Table 1).

**Table 1:** Child and Maternal Characteristics by Prior Child Protective Services (CPS) History (*n* = 727)

	Prior CPS History (*n* = 312)		No Prior CPS History (*n* = 415)		Total (*n* = 727)	
	Mean (SD)	Range	Mean (SD)	Range	Mean (SD)	Range
Child’s Age	8.1 (4.0)	0.2-17.8	7.7 (4.0)	0.5-17.3	7.9 (4.0)	0.2-17.8
Mother’s Age	34.2 (8.6)	14.0-63.0	33.8 (7.7)	14.0-63.0	34.0 (8.1)	14.0-64.3
	n	%	n	%	n	%
Child’s Gender						
Male	107	43.5	139	56.5	246	33.8
Female	205	42.6	276	57.4	481	66.2
Race/Ethnicity*						
White	12	21.1	45	79.0	57	7.8
Black	122	56.7	93	43.3	215	29.6
Hispanic	149	38.9	234	61.1	383	52.7
Other	29	40.3	43	59.7	72	9.9
Mother’s Primary Language*						
English	241	49.5	246	50.5	487	67.0
Spanish	45	28.9	111	71.2	156	21.5
Other	26	31.0	58	69.0	84	11.6
Mother’s Education Level*						
< HS Grad	54	41.9	75	58.1	129	17.7
HS Graduate	138	48.9	144	51.1	282	38.8
Some College/ College Grad	120	38.0	196	62.0	316	43.5
Public Assistance*	170	50.3	168	49.7	338	46.5
Individual adverse items						
Domestic Abuse*	168	51.9	156	48.2	324	44.6
Problems with Police*	78	61.9	48	38.1	126	17.3
Drug/Alcohol Problem*	34	77.3	10	22.7	44	6.1
Mental health Problems*	114	59.1	79	40.9	193	26.6
Ongoing Custody Battle*	50	61.0	32	39.0	82	11.3
Adversity Category*						
None	73	27.2	203	72.8	279	38.4
One	96	41.9	133	58.1	229	31.5
Two or more	140	63.9	79	36.1	219	30.1

*p < 0.05 comparing covariates and prior CPS history

In binomial regression analyses, the greater the number of adversities, the more likely a history of prior CPS report (experiencing one adversity [Prevalence Ratio (PR) 1.25 95%CI 1.04, 1.50 or 2 or more adversities PR 1.67 95%CI 1.41, 1.98], even after adjusting for socio-demographic factors. (Table 2).

**Table 2:** Binomial Regression Models of the Childhood Adversities and prior Child Protective Service (CPS) History (*N* = 727)

	Unadjusted Model	Adjusted Model^a^
	PR [95% CI]	PR [95% CI]
Adversity Category		
None	Ref	Ref
One	1.57 (1.20–2.00]^a^	1.25 [1.04–1.50]^a^
Two or more	2.36 [1.89–2.91]^a^	1.67 [1.41–1.98] ^a^

^a^*p*-value < 0.05

^a^Model Adjusted for child gender, race/ethnicity, age, type of abuse, maternal age, primary language spoken at home, education level, and receipt of public assistance

## Discussion

It has been suggested that pediatricians could support CPS- involved families with close follow-up and referrals to appropriate services in the community (Campbell et al. 2012). Child abuse pediatricians can do just that within their CACs’ role: assessing adverse conditions for each family seen, connecting families to community based services in collaboration with the multidisciplinary team, and when possible involve the child’s pediatrician in the overall treatment plan of the family. Coordinated and integrated collaboration between primary care providers and investigative teams will ensure the delivery of needed services at the community level for families for whom adversities are known (Campbell et al. 2019; Bair-Merritt and Zuckerman 2016).

We acknowledge adversities were self-reported by the mother in the setting of a child abuse investigation and some were not assessed with validated scales. While the CAC population does not reflect the entire CPS population, our study findings provide further evidence that maltreated children are exposed to a host of other family dysfunction factors, addressing adversities at the first encounter of suspicion for child abuse may prevent further victimization of children. In addition, our study further supports the need to support a family in the face of an investigation by assessing and addressing other adversities; encouraging collection of data with more accurate measures of adversities, by using for example, documented domestic violence reports or prior child abuse reports and presenting an opportunity to study new models that integrate multiple social factors to build safe, stable and nurturing relations for children (Sege et al. 2017).

## Data Availability

Please contact author for data requests.
